# Ultra-High Prostate-Specific Antigen Level: A Potential Very-High-Risk Factor for Localized High-Risk Prostate Cancer

**DOI:** 10.3390/cancers15235644

**Published:** 2023-11-29

**Authors:** Hideya Yamazaki, Gen Suzuki, Koji Masui, Kei Yamada, Takashi Ueda, Takumi Shiraishi, Atsuko Fujihara, Takashi Kato, Yasutoshi Hashimoto, Haruumi Okabe

**Affiliations:** 1 Department of Radiology, Graduate School of Medical Science, Kyoto Prefectural University of Medicine, 465 Kajiicho Kawaramachi Hirokoji, Kamigyo-ku, Kyoto 602-8566, Japan; gensuzu@koto.kpu-m.ac.jp (G.S.); mc0515kj@koto.kpu-m.ac.jp (K.M.); kyamada@koto.kpu-m.ac.jp (K.Y.); 2 Department of Urology, Graduate School of Medical Science, Kyoto Prefectural University of Medicine, 465 Kajiicho Kawaramachi Hirokoji, Kamigyo-ku, Kyoto 602-8566, Japan; t-ueda@koto.kpu-m.ac.jp (T.U.); takumi14@koto.kpu-m.ac.jp (T.S.); fujihara@koto.kpu-m.ac.jp (A.F.); 3 Department of Radiology, Ujitakeda Hospital, Uji-City 611-0021, Kyoto, Japan; hymed_housya@yahoo.co.jp (T.K.); yasu06340829@yahoo.co.jp (Y.H.); h-okabe@takedahp.or.jp (H.O.)

**Keywords:** prostate cancer, brachytherapy, intensity modulated radiotherapy, stereotactic body radiotherapy, prostate-specific antigen (PSA)

## Abstract

**Simple Summary:**

NCCN risk classifications for prostate cancer do not include elevated initial PSA levels as a single very-high-risk factor. However, an ultra-high initial PSA level (>50 ng/mL) showed a similar hazard ratio in the biochemical disease-free survival rate and the distant metastasis-free survival rate to the other single very-high-risk factors of T3b–4 and a Gleason score of 9–10. Therefore, an ultra-high initial PSA level has the potential to be a single very-high-risk factor for localized prostate cancer.

**Abstract:**

To examine the impact of ultra-high iPSA levels of >50 ng/mL (uhPSA) after modern radiotherapy, we compared outcomes of 214 patients with uhPSA levels to 1161 other high-risk patients. Radiotherapy included brachytherapy ± external beam radiotherapy (EBRT) and EBRT alone (intensity-modulated radiotherapy or stereotactic body radiotherapy). The biochemical disease-free survival rate (bDFS), the distant metastasis-free survival rate (DMFS), local control, and pelvic lymph node control were analyzed. Patients with uhPSA levels had an inferior bDFS (84.8% at 5 years) and DMFS (93.9% at 5 years) compared to other high-risk patients (92.7% and 97.2%, both *p* < 0.001). The uhPSA group showed more distant metastases than the non-uhPSA group; however, the frequencies of local failure and pelvic lymph node recurrence were similar. The uhPSA group demonstrated hazard ratios (HRs) of 2.74 for bDFS and 2.71 for DMFS, similar to those of T3b–4 (HR 2.805 and 2.678 for bDFS and DMFS) and GS 9–10 (HR 2.280 and 2.743 for bDFS and DMFS). An uhPSA level could be a candidate for a single VHR factor to identify high-risk patients who require intensified treatment.

## 1. Introduction

Prostate cancer is the most commonly diagnosed malignancy in men in Western countries [1,2]. In 2021, approximately 248,530 new cases of prostate cancer were diagnosed in the United States, accounting for 10.7% of cancer-related deaths [2]. For classification, we used several risk stratifications. The National Comprehensive Cancer Network (NCCN) risk classification is well known and is the most utilized risk classification system worldwide [2]. Recently, they further subdivided risk stratification into meticulously detailed classifications: very low, low, intermediate, high, and very high risk (VHR), with VHR being the category of patients with the worst prognosis [2]. The VHR group includes the presence of any of the following disease characteristics: cT3b–4, multiple NCCN high-risk factors, primary Gleason pattern 5 disease and/or ≥5 biopsy cores with Gleason scores (GSs) of 8–10.

Although these criteria are useful, the initial prostate-specific antigen (iPSA) level is not included as a single VHR factor, despite GS 9–10 and T3b–4 being included. This is partly because a VHR was determined in patients undergoing surgery who rarely showed a very high level of PSA [3]. In addition, Rodrigues et al. found that a very high level of PSA was found to be a significant factor for risk of distant metastasis (HR 1.01 95% CI 1.001–1.02) but not the biochemical disease-free survival rate (bDFS) [4]. As that report highlights the increased risk of systemic dissemination in patients with a very high level of PSA, there was an opinion that patients with very high levels of PSA were not good candidates for local therapy [3,4]. Then, patients with very high levels of PSA were often underrepresented or excluded from randomized clinical trials [4]. Consequently, little is known about their optimal treatment and prognosis [4,5,6,7,8]. Therefore, we examined the role of ultra-high iPSA levels of >50 ng/mL (uhPSA) following modern radiotherapy.

To create a large cohort, we used freely available public data on patients undergoing radiotherapy, including high-dose-rate brachytherapy (HDR-BT), external beam radiotherapy (EBRT), intensity-modulated radiotherapy (IMRT), and stereotactic body radiotherapy (SBRT) [9,10,11]. We combined data from patients treated with low-dose-rate brachytherapy (LDR-BT) with or without EBRT [12] and IMRT [13] at our institutions. We aimed to determine the role of uhPSA as a single, independent VHR factor of prognosis for patients with localized prostate cancer treated with radiotherapy.

## 2. Materials and Methods

### 2.1. Patients

Patients treated with modern EBRT (IMRT and SBRT, *n* = 420) and BT with or without EBRT (*n* = 955) were retrospectively reviewed. We used data of public databases including 916 patients treated with HDR-BT, 67 treated with SBRT, and 206 treated with IMRT [9,10,11]. We also used data of 39 patients treated with LDR-BT at Kyoto Prefectural University of Medicine [12] and 147 patients treated with IMRT at Uji Takeda Hospital [13]. We included patients with histologically and clinically confirmed T1 to T4N0M0 prostate cancer with GS and iPSA data that allowed risk classification to be determined according to the NCCN [2]. The endpoints are biochemical disease-free survival (bDFS), distant metastasis-free survival (DMFS), prostate cancer-specific mortality (PCSM), and overall survival (OS). Biochemical failure was defined according to the Phoenix ASTRO consensus (nadir + 2 ng/mL). Those endpoints were defined as the interval from the first day of radiotherapy to PSA failure, development of distant metastases, PCSM, or all causes of death. Clinical recurrence includes local recurrence, pelvic lymph node recurrence, and distant metastases. Patients with imaging findings confirming clinically or pathologically diagnosed metastatic disease were classified as having clinical recurrence. PCSM was defined if prostate cancer being recorded as the primary cause of death. The institutional review board of each participating center approved the study protocol for the construction of the public database [9,10,11]. All patients included in the analysis from Kyoto Prefectural University of Medicine and Uji Takeda Hospital provided written informed consent. This study was conducted in accordance with the principles of the Declaration of Helsinki and was approved by the Kyoto Prefectural University of Medicine Institutional Review Board (ERB-C-1403).

### 2.2. Treatment

EBRT included IMRT and SBRT; 353 patients who had been treated with IMRT and 67 treated with SBRT using >70 Gy in equivalent doses of 2 Gy fractions (EQD2Gy) (*n* × d ([α/β] + d)/([α/β] + 2), where *n* = number of treatment fractions; d = dose per fraction in Gy, α/β = 1.5 Gy) were included because dose escalation has been shown to improve biochemical control in patients with localized prostate cancer, and the NCCN guidelines recommend doses of >70 Gy in conventional fractions [2].

The details of the treatment schedules are shown in Supplementary Table S1. A total of 206 IMRT data were obtained from a freely accessible dataset [9], and 147 image-guided IMRTs using helical tomotherapy were performed at the Department of Radiology at Uji Takeda Hospital [13]. Major IMRT treatment schedules were as follows: 74 Gy/37 fractions (*n* = 82), 74.8 Gy/34 fractions (*n* = 65), 70 Gy/28 fractions (*n* = 46), 78 Gy/39 fractions (*n* = 50), 80 Gy/40 fractions (*n* = 34), 72 Gy/36 fractions (*n* = 70). The details of the treatment procedure of IMRT have been described elsewhere [13], and the median prescribed dose was 74 Gy (range, 62–80 Gy) in 36 fractions (range, 20–40 fractions). For SBRT, the median prescribed SBRT dose was 36 Gy (range, 32–36 Gy) in four fractions (range, 4–5 fractions) (Supplementary Table S1) [10].

BT includes HDR-BT (*n* = 916) and LDR-BT (*n* = 39). Details of the HDR-BT treatment plan for the HDR and EBRT groups are shown in Supplementary Table S1 [10,12]. The main regimens of HDR-BT were: HDR-BT 31.5 Gy/5 times + EBRT 30 Gy/10 times (*n* = 561), HDR-BT 18 Gy/2 times (*n* = 208) plus EBRT 39 Gy/13 fraction or 51 Gy/17 fraction or 48 Gy/16 fraction, HDR-BT 11 Gy/1 fraction and EBRT 51 Gy/17 fraction (*n* = 103), HDR-BT 21 Gy/2 fraction or HDR-BT 21 Gy/3 fraction (*n* = 28) and EBRT 45 Gy/15 fraction, or EBRT 42 Gy/14 fraction, or EBRT 51 Gy/17 fraction, HDR-BT 20 Gy/2 fraction (*n* = 10) Plus EBRT 46 Gy/23 fractions or EBRT 30 Gy/15 fractions. The average HDR-BT dose was 31.5 Gy (range 10.5–31.5 Gy) and the average fraction size with additional EBRT in different fractions was 6.3 Gy (range 5–11 Gy) (average dose 31.5 Gy; medium fraction). Size: 3 Gy; Range: 2–3 Gy. Considering LDR-BT with or without EBRT, we used a prescribed dose of 145 Gy (GS ≤ 6) or 110 Gy (GS ≥ 7, combined with 40 Gy/20 fractions of EBRT) [12].

### 2.3. Statistical Analyses

A Fisher’s exact test was used for percentage comparisons, and the Mann–Whitney U test was used to compare means or medians. The Kaplan–Meier method was used to analyze survival data (bDFS, DMFS, PCSM, and OS) and was compared using the log-rank test. A cause-specific approach (death due to another cause of cancer was assigned as a censor) was applied to bDFS, DMFS, and PCSM. For estimating hazard ratios (HRs), Cox’s proportional hazard model was used for univariate and multivariate analyses of bDFS, DMFS, PCSM, and OS. For the multivariate analysis of bDFS and DMFS, the following factors were evaluated: BT vs. EBRT, age (≤74 years vs. ≥75 years), T category (T1-3a vs. T3b-4), iPSA (≤50 ng/mL vs. >50 ng/mL), and GS (≤8 or ≥9). Androgen deprivation therapy (ADT) use was excluded from the multivariate analysis because of the small number of patients who did not receive ADT. *p* values and 95% confidence intervals (CIs) were calculated, and all results were considered significant if the *p* value was <0.05. All statistical analyses were performed using StatView 5.0 and EZR statistical software Version 1.52 [14].

## 3. Results

### 3.1. Patients and Treatment Characteristics

The patient characteristics are shown in Table 1. The median patient age was 70.6 years (range: 48–89 years) and the median follow-up period for the entire cohort was 61 months (range: 2–177 months), with a 1-year minimum for surviving patients or until death. Table 1 compares the patient background characteristics of the two groups (an iPSA of >50 ng/mL defined the uhPSA group and an iPSA of 50 or less defined the non-uhPSA group).

The uhPSA group included patients with advanced disease who required more hormonal therapy than the non-uhPSA group did.

### 3.2. Assessment of bDFS, DMFS, PCSS, and OS According to iPSA Level

Overall, 42 (19.6%) uhPSA patients developed biochemical failure compared with 83 (7.1%) patients in the non-uhPSA group (*p* < 0.001; Table 2).

The uhPSA group showed a significantly lower bDFS than the non-uhPSA group.

**Figure 1:**
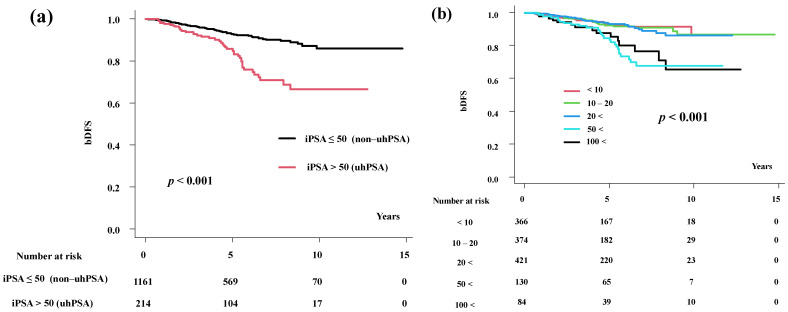
Biochemical disease-free survival. (**a**) Biochemical disease-free survival rates according to iPSA level. (**b**) Biochemical disease-free survival rates according to detailed iPSA level.

The actuarial 5-year bDFS values were 92.7% (95% confidential interval (95% CI), 90.7–94.2%) and 84.8% (95% CI, 78.3–89.5%) (*p* < 0.001, Figure 1a) in the non-uhPSA and uhPSA groups, respectively.

In a detailed analysis, we found that the iPSA < 10, iPSA 10–20, and iPSA 20–50 groups showed a similar bDFS, and the iPSA < 50 and iPSA < 100 groups also demonstrated a similar bDFS. These figures were 92.8%, 92.2%, 92.9%, 83.3%, and 87.4% (*p* < 0.001, Figure 1b) in the iPSA < 10 ng/mL, 10–20 ng/mL, 20–50 ng/mL, 50–100 ng/mL, and >100 ng/mL groups, respectively.

The uhPSA group showed a significantly higher clinical failure ratio (9.3%) than the non-uhPSA group (3.7%). The uhPSA group had more distant metastases than the non-uhPSA group; however, the frequencies of local failure and pelvic lymph node recurrence were similar (Table 2; *p* = 0.001).

**Figure 2:**
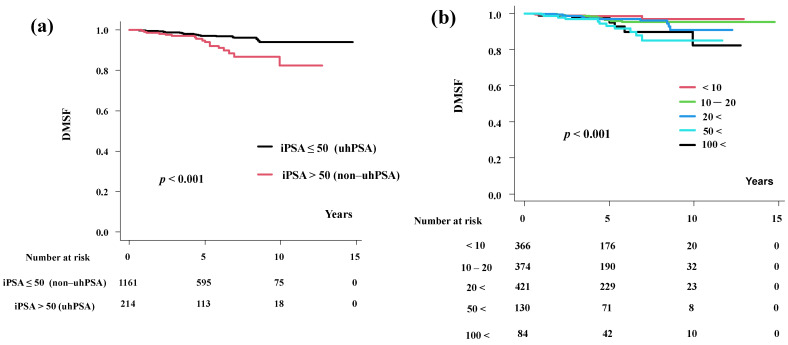
Distant metastasis-free survival. (**a**) Distant metastasis-free survival rates according to iPSA level. (**b**) Distant metastasis-free survival rates according to detailed iPSA level.

Fifty patients had distant metastases (non-uhPSA group, *n* = 33; uhPSA group, *n* = 17, *p* = 0.001, Table 2), and the 5-year DMFS was 97.2% (95.8–98.1%) in non-uhPSA group and 93.9% (88.7–96.7%, *p* < 0.001) in the uhPSA group (Figure 2a). There was a significant difference in the DMFS between the non-uhPSA and uhPSA groups. In detail, these figures were 98.5%, 96.3%, 96.9%, 93.1%, and 95.1% (*p* < 0.001, Figure 2b) in iPSA < 10 ng/mL, 10–20 ng/mL, 20–50 ng/mL, 50–100 ng/mL, and >100 ng/mL groups, respectively.

No statistically significant difference was found in PCSM between uhPSA and non-uhPSA groups. The 5-year PCSM values were 99.3% (98.4–99.7%) and 98.5% (94.1–99.6%, *p* = 0.387) in the non-uhPSA and uhPSA groups, respectively.

The uhPSA group showed a significantly lower OS than the non-uhPSA group. The 5-year OS rates were 97.3% (96.0–98.2%) and 92.2% (86.8–95.4%, *p* = 0.042) in the non-uhPSA and uhPSA groups, respectively.

### 3.3. Correlation and Comparison among VHR Factors

**Figure 3:**
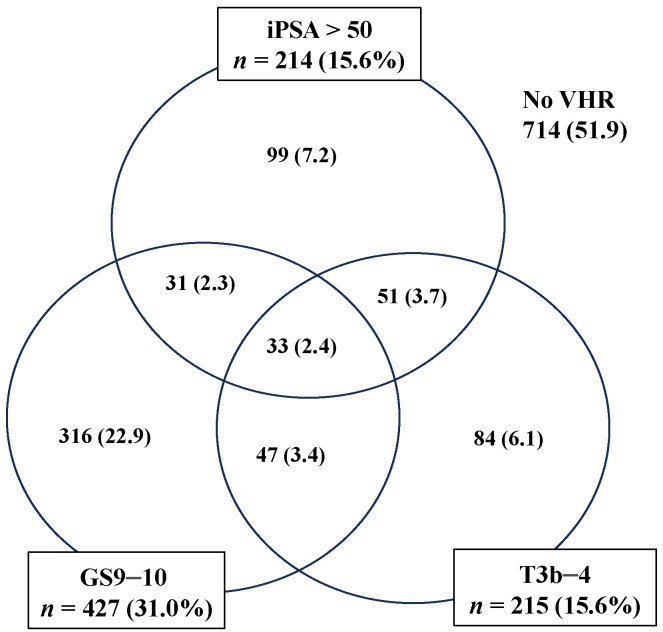
Distribution of subgroup according to very-high-risk factors. % in parenthesis.

The correlations and characteristics of each VHR factor (T3b–4, GS 9–10, and uhPSA) and the combination of these VHR factors are shown in Figure 3.

The univariate analysis for each VHR factor and subgroup is shown in Table 3. uhPSA showed similar HRs to the other two VHR factors (uhPSA HR 2.35, T3b–4, HR 2.05 and GS 9–10, HR 2.27) for bDFS and DMFS (uhPSA, HR 2.23; T3b–4, HR 2.05; GS 9–10, HR 2.96). In the subgroup analysis, GS 9–10 + uhPSA (HR 4.438 for bDFS, HR 6.139 for DMFS) and GS 9–10 + T3b–4 (HR 4.985 for bDFS, HR 7.503 for DMFS) showed the highest HR.

**Figure 4:**
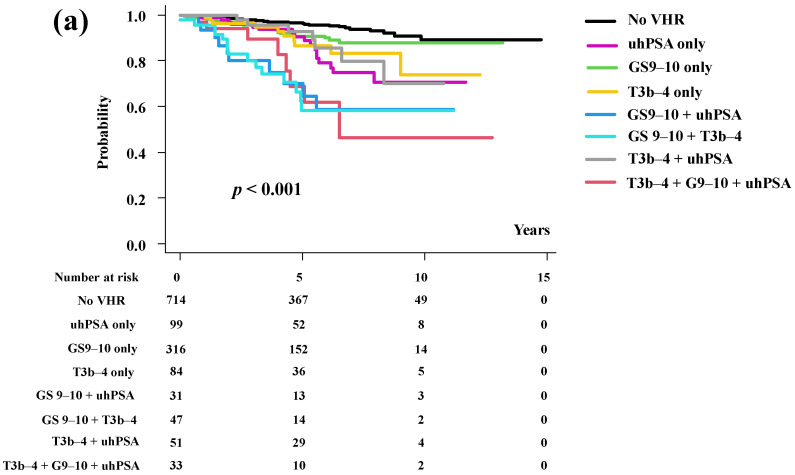

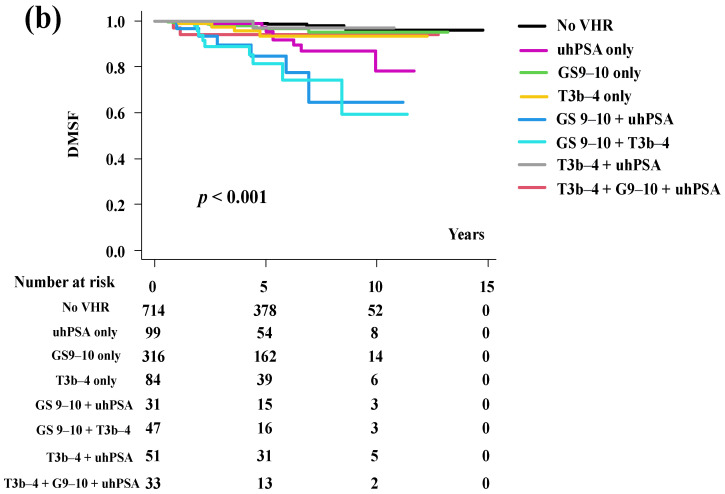
Biochemical disease-free survival and distant metastasis-free survival in the subgroup analysis. (**a**) Biochemical disease-free survival among subgroups. (**b**) Distant metastasis-free survival among subgroups.

The actuarial 5-year bDFS was 96.3%, 90.9%, 90.4%, 86.5%, 58.3%, 64.6%, 92.7%, and 68.8% (*p* < 0.001, Figure 4a) in the No VHR, GS 9–10 only, uhPSA only, T3b–4 only, GS 9–10 + T3b–4, GS 9–10 + uhPSA, T3b–4 + uhPSA, and all three VHR (uhPSA + T3b–4 + GS 9–10) subgroups, respectively.

GS 9–10 + uhPSA and GS 9–10 + T3b–4 groups showed the worst DMFS.

The actuarial 5-year DMFS was 98.8%, 96.8%, 95.4%, 93.3%, 81.5%, 84.5%, 97.1%, and 93.9% (*p* < 0.001, Figure 4b) in the No VHR, GS 9–10 only, uhPSA only, T3b–4 only, GS 9–10 + T3b–4, GS 9–10 + uhPSA, T3b–4 + uhPSA, and all three VHR (uhPSA + T3b–4 + GS 9–10) subgroups, respectively.

In multivariate analyses, GS 9–10, uhPSA and T3b–4 were the significant predictors of a bDFS with HRs of 2.27, 2.35, and 2.05 (Table 4). For DMFS, the BT usage, GS 9–10, uhPSA, and T3b–4 were significant predictors, with HRs of 0.28, 2.96, 2.23, and 2.05 (Table 4).

## 4. Discussion

We determined the role of uhPSA in the outcomes of patients with localized prostate cancer treated with radiotherapy. We found that uhPSA is an important and strong predictor not only of PSA control but also of DMFS, with a comparably high HR for the already established VHR factors T3b–4 and GS 9–10. This finding is consistent with those of other studies, showing that elevated iPSA levels at the time of diagnosis are highly predictive of metastatic disease following either radiotherapy or surgery [3,4,5,6,7]. Our data suggest that future modifications to the NCCN guidelines should consider elevated iPSA levels as a single criterion for inclusion in the VHR group.

Sundi et al. defined the VHR group based on surgical outcomes [3]. The 2014 NCCN guidelines were revised according to these data and the presence of primary Gleason grade 5 or ≥5 scores with GS 8–10 was added as a new criterion for inclusion into the VHR group. Narang et al. confirmed the role of VHR factors in a cohort of patients treated with radiotherapy between 1993 and 2006 [15]. However, this confirmatory study was limited by the use of conventional radiation techniques that do not reflect modern radiotherapy. The strength of our data is the inclusion of patients treated with radiotherapy using dose-escalated modern radiotherapy techniques.

Notably, the group with iPSA 20–50 ng/mL was not significantly different from the iPSA less than 20 ng/mL group, which may partly be a reason why the iPSA level could not incorporated as a VHR factor, because previous outcome analyses for VHR were conducted on results from surgical procedures which may exclude an uhPSA of ≥100 ng/mL. Accordingly, some authors have proposed that the uhPSA group is not a good candidate for local therapy and instead is a candidate for systemic therapy, partly because elevated PSA levels imply the existence of disease outside the prostate [16,17,18]. Our data partially concur with this opinion, because the uhPSA group showed increased PSA failure with more distant metastases, but not elevated local failure nor pelvic lymph node recurrence. However, recent trends have enhanced the role of local radiotherapy, which is beneficial even in cases of distant metastases with a hormone-sensitive, low metastatic burden [19,20]. Guarneri et al. reported that patients with high iPSA levels (≥20 ng/mL) showed favorable clinical outcomes, supporting the role of local radiotherapy as the primary therapy in combination with long-term ADT for patients with high PSA levels at diagnosis [7].

The combination of VHR factors was also beneficial for identifying high-risk groups. We found that GS 9–10 + uhPSA (HR 4.438 for bDFS, HR 6.139 for DMFS), GS 9–10 + T3b–4 (HR 4.985 for bDFS, HR 7.503 for DMFS), and GS 9–10 + uhPSA + T3b–4 (HR 3.855 for bDFS, HR 2.134 for DMFS) had the worst prognoses for bDFS and DMFS. In contrast, a single VHR factor without other VHR factors (uhPSA only: HR 1.845 for bDFS and HR 2.182 for DMFS; T3b–4 only: HR 1.386 for bDFS and HR 1.350 for DMFS; GS 9–10 only: HR 0.938 for bDFS and HR 0.803 for DMFS) showed a slightly worse prognosis.

Advanced novel imaging techniques, such as multiparametric magnetic resonance imaging (mpMRI) [21] and positron emission tomography with prostate-specific membrane antigen ligands (PSMA-PET), have been incorporated recently. mpMRI provides detailed anatomical information to identify the location and size of tumors within the prostate [22]. PSMA-PET imaging allows for the more accurate assessment of disease spread, especially in the lymph nodes and bones. These early and precise detection techniques can influence treatment decisions, potentially allowing for a more targeted and aggressive approach. Utilizing these imaging techniques; intensive treatments, including whole pelvic radiotherapy; escalated-dose radiotherapy, including boost radiotherapy using BT or IMRT [23,24,25]; and new drugs (abiraterone, etc.) [26,27,28] would be beneficial for the VHR group, including the uhPSA group.

This study has some limitations. First, this was a retrospective, multi-institutional study with substantial heterogeneity. Therefore, studies with longer follow-up periods and larger patient cohorts are required to obtain more accurate results. In particular, a longer follow-up period is required to examine PCSM and OS because there were only a few events to estimate the PCSM and OS. This is partly because of the good prognosis of Japanese patients who respond well to ADT, which could mask the efficacy of radiotherapy [29]. In addition, Zumsteg et al. reported that there is a need for a long time to progress, with the median times to metastasis and death from PSA failure reported to be 5.4 and 10.5 years, respectively [30]. Second, the heterogeneity in pathological examinations is a limitation. The biopsy method is evolving with the advancement of multiparametric magnetic resonance imaging, and a central pathology review could be useful for evaluating Gleason grading and the number of positive cores used in VHR classification in the NCCN, because it varies between institutions.

Despite these limitations, to the best of our knowledge, this is the first and largest comprehensive study to analyze the role of uhPSA in modern radiotherapy outcomes in patients with localized prostate cancer. Our findings may be beneficial for counseling patients with uhPSA prostate cancer regarding treatment and prognosis.

## 5. Conclusions

Patients with uhPSA levels exhibited worse prognoses and clinical outcomes than the non-uhPSA group, which was comparable to other single VHR factors (GS 9–10 and T3b–4 categories). uhPSA levels could be a candidate for a single VHR factor that can identify high-risk patients who require intensified treatment.

## Figures and Tables

**Table 1:** Background patient characteristics.

Variables	Strata	Total	iPSA ≤ 50 (Non-uhPSA)	iPSA > 50 (uhPSA)	*p*-Value
		No. (%) or Median [Range]	No. (%) or Median [Range]	No. (%) or Median [Range]	
Age		71.00 [48.00, 89.00]	71.00 [48.00, 86.00]	70.00 [51.50, 89.00]	**0.015**
initial PSA	(ng/mL)	17.90 [2.68, 1454.00]	14.40 [2.68, 50.00]	83.14 [50.20, 1454.00]	**<0.001**
Gleason score sum	6	99 (7.2)	93 (8.0)	6 (2.8)	**0.024**
	7	476 (34.6)	392 (33.8)	84 (39.3)	
	8	371 (27.0)	312 (26.9)	59 (27.6)	
	9–10	429 (31.2)	364 (31.4)	65 (30.4)	
T category	T1–2	635 (46.2)	594 (51.2)	41 (19.2)	**<0.001**
	T3a	525 (38.2)	436 (37.6)	89 (41.6)	
	T3b–4	215 (15.6)	131 (11.3)	84 (39.3)	
Modality	BT ± EBRT	955 (69.5)	805 (69.3)	150 (70.1)	0.872
	EBRT	420 (30.5)	356 (30.7)	64 (29.9)	
ADT	No	87 (6.3)	85 (7.3)	2 (0.9)	**<0.001**
	Yes	1288 (93.7)	1076 (92.7)	212 (99.1)	

BT; brachytherapy, EBRT; external beam radiotherapy, uhPSA; ultra-high PSA level, ADT; androgen deprivation therapy. *p*-value was calculated between iPSA ≤ 50 (non-uhPSA) and iPSA > 50 (uhPSA). Bold values indicate statistically significance.

**Table 2:** Outcomes of radiotherapy according to iPSA level.

Variables	Strata	Total	iPSA ≤ 50 (Non-uhPSA)	iPSA > 50 (uhPSA)	*p*-Value
		No. (%)	No. (%)	No. (%)	
PSA failure	No	1250 (90.9)	1078 (92.9)	172 (80.4)	**<0.001**
	Yes	125 (9.1)	83 (7.1)	42 (19.6)	
Clinical recurrence	No	1312 (95.4)	1118 (96.3)	194 (90.7)	**0.001**
	Yes	63 (4.6)	43 (3.7)	20 (9.3)	
Local Failure	No	2483 (99.5)	1154 (99.4)	213 (99.5)	1
	Yes	12 (0.5)	7 (0.6)	1 (0.5)	
Pelvic lymph node metastasis	No	1357 (98.7)	1146 (98.7)	211 (98.6)	0.752
	Yes	18 (1.3)	15 (1.3)	3 (1.4)	
Distant metastasis	No	1325 (96.4)	1128 (97.2)	197 (92.1)	**0.001**
	Yes	50 (3.6)	33 (2.8)	17 (7.9)	
Prostate cancer related mortality	Alive	1359 (98.8)	1149 (99.0)	210 (98.1)	0.295
	Death	16 (1.2)	12 (1.0)	4 (1.9)	
Overall survival	Alive	1311 (95.3)	1114 (96.0)	197 (92.1)	**0.02**
	Death	64 (4.7)	47 (4.0)	17 (7.9)	

*p*-value was calculated between iPSA ≤ 50 (non-uhPSA) and iPSA > 50 (uhPSA). Bold values indicate statistically significance.

**Table 3:** Univariate analysis for PSA control and distant metastasis-free survival ratio among tested VHRS.

Tested VHR Definitions	bDFS	*p*-Value	DMFS	*p*-Value	
	Hazard Ratio (95% CI)		Hazard Ratio (95% CI)		Sample Size (%)
T category: T3b–4	2.805 (1.926–4.086)	**<0.001**	2.678 (1.477–4.853)	**0.001**	215 (15.6)
Gleason score sum: GS9–10	2.280 (1.604–3.242)	**<0.001**	2.743 (1.572–4.784)	**<0.001**	427 (31.0)
iPSA: iPSA > 50 (uhPSA)	2.741 (1.891–3.974)	**<0.001**	2.656 (1.479–4.771)	**0.001**	214 (15.6)
**Subgroup**					
uhPSA only	1.845 (1.106–3.077)	0.018	2.182 (1.023–4.651)	**0.043**	99 (7.2)
GS 9–10 only	0.938 (0.609–1.446)	0.773	0.803 (0.389–1.654)	0.552	316 (22.9)
T3b–4 only	1.386 (0.726–2.645)	0.322	1.350 (0.485–3.752)	0.565	84 (6.1)
GS 9–10 + uhPSA	4.438 (2.325–8.471)	**<0.001**	6.139 (2.615–14.41)	**<0.001**	31 (2.3)
GS 9–10 + T3b–4	4.985 (2.903–8.56)	**<0.001**	7.503 (3.641–15.46)	**<0.001**	47 (3.4)
T3b–4 + uhPSA	1.413 (0.6592–3.03)	0.374	0.475 (0.065–3.441)	0.461	51 (3.7)
T3b–4 + GS9–10 + uhPSA	3.855 (1.881–7.90)	**<0.001**	2.134 (0.518–8.787)	0.293	33 (2.4)

GS 9–10 + uhPSA, GS 9–10 + T3b–4, and all three VHR groups showed the worst bDFS. Bold values indicate statistically significance.

**Table 4:** Multivariate analysis for predicator of PSA control and distant metastasis-free survival rates.

Factor	Strata	bDFS	*p*-Value	DMFS	*p*-Value
		Hazard Ratio (95% CI)		Hazard Ratio (95% CI)	
Age	–74 vs. 75–	1.12 (0.74–1.69)	0.61	1.10 (0.55–2.24)	0.78
Modality	BT vs. EBRT	1.33 (0.89–1.96)	0.16	0.28 (0.11–0.72)	**0.008**
Gleason score sum	–8 vs. 9–10	2.27 (1.59–3.26)	**<0.001**	2.96 (1.69–5.18)	**<0.001**
iPSA	–50 (non-uhPSA) vs. <50 (uhPSA)	2.35 (1.56–3.53)	**<0.001**	2.23 (1.18–4.21)	**0.013**
T category	–3a vs. 3b–4	2.05 (1.36–3.10)	**<0.001**	2.05 (1.08–3.90)	**0.028**

Abbreviations: uhPSA: ultra-high level of iPSA, bDFS: biochemical disease-free survival, DMFS: distant metastasis-free survival. Bold values indicate statistically significance.

## Data Availability

The data of this study can be obtained from the author upon reasonable request.

## Supplementary Materials

The following supporting information can be downloaded at: https://www.mdpi.com/article/10.3390/cancers15235644/s1, Table S1, Detailed treatment schedule.
